# Evaluating predictive modeling algorithms to assess patient eligibility for clinical trials from routine data

**DOI:** 10.1186/1472-6947-13-134

**Published:** 2013-12-09

**Authors:** Felix Köpcke, Dorota Lubgan, Rainer Fietkau, Axel Scholler, Carla Nau, Michael Stürzl, Roland Croner, Hans-Ulrich Prokosch, Dennis Toddenroth

**Affiliations:** 1 Chair of Medical Informatics at the University Erlangen-Nuremberg, Krankenhausstraße 12, 91054 Erlangen, Germany; 2 Department of Radiation Oncology, Erlangen University Hospital, Erlangen, Germany; 3 Department of Anesthesiology, Erlangen University Hospital, Erlangen, Germany; 4 Division of Molecular and Experimental Surgery, Erlangen University Hospital, Erlangen, Germany; 5 Department of Surgery, Erlangen University Hospital, Erlangen, Germany

## Abstract

**Background:**

The necessity to translate eligibility criteria from free text into decision rules that are compatible with data from the electronic health record (EHR) constitutes the main challenge when developing and deploying clinical trial recruitment support systems. Recruitment decisions based on case-based reasoning, i.e. using past cases rather than explicit rules, could dispense with the need for translating eligibility criteria and could also be implemented largely independently from the terminology of the EHR’s database. We evaluated the feasibility of predictive modeling to assess the eligibility of patients for clinical trials and report on a prototype’s performance for different system configurations.

**Methods:**

The prototype worked by using existing basic patient data of manually assessed eligible and ineligible patients to induce prediction models. Performance was measured retrospectively for three clinical trials by plotting receiver operating characteristic curves and comparing the area under the curve (ROC-AUC) for different prediction algorithms, different sizes of the learning set and different numbers and aggregation levels of the patient attributes.

**Results:**

Random forests were generally among the best performing models with a maximum ROC-AUC of 0.81 (CI: 0.72-0.88) for trial A, 0.96 (CI: 0.95-0.97) for trial B and 0.99 (CI: 0.98-0.99) for trial C. The full potential of this algorithm was reached after learning from approximately 200 manually screened patients (eligible and ineligible). Neither block- nor category-level aggregation of diagnosis and procedure codes influenced the algorithms’ performance substantially.

**Conclusions:**

Our results indicate that predictive modeling is a feasible approach to support patient recruitment into clinical trials. Its major advantages over the commonly applied rule-based systems are its independency from the concrete representation of eligibility criteria and EHR data and its potential for automation.

## Background

Over the decades, the number of participants in numerous clinical trials has frequently fallen short of expectations [1-3]. When discrepancies between planned and actual recruitment rates occur, trials need to be prolonged at considerable cost or may even have to be aborted [4]. The advent of electronic health records (EHR) and the increasing volume of medical data contained within them have raised the question whether and how these data can be reused to improve the recruitment process of clinical trials. In a recent literature review Cuggia et al. identified 28 different clinical trial recruitment support systems (CTRSS) and found that *’all systems require the input of both patient data and eligibility criteria’*[5]. The core functionality of all CTRSS is the comparison of these two inputs to evaluate whether a given patient suits the trial.

Unfortunately, trial protocols conventionally describe eligibility criteria in free text, which cannot be directly applied to data in the EHR’s database. These criteria therefore have to be encoded into a set of EHR-compatible rules, a process which has proven difficult in practice. In an analysis of 1000 randomly selected eligibility criteria, Ross et al. [6] classified 85% of the criteria as semantically complex. They conclude that ‘*researchers trying to determine patient eligibility for studies face incomprehensible and ambiguous criteria as well as under-specified criteria requiring clinical judgment or assessments.’*

Weng et al. [7] found 27 different languages proposed to represent eligibility rules in a computable format. They distinguish ad hoc expressions, Arden Syntax, logic-based languages, object-oriented query languages and temporal query languages. More expressive languages can cover the original meaning of the eligibility criteria, but become more complex at the same time, resulting in a more tedious translation process. Still, none of the languages seems to be able to accurately represent all eligibility criteria. Tu et al. [8] estimate up to 60% criteria coverage for the ad hoc expression language ERGO. Wang et al. [9] estimate that their advanced Arden Syntax is able to describe up to 90% of all eligibility criteria. While complex languages for eligibility rules representation have advantages in terms of criteria coverage, they often rely on IT systems that are uncommon in general practice and require a high level of expertise. That this seems to limit adoption by other clinics is underlined by the fact, that most CTRSS so far relied on SQL statements to represent eligibility criteria.

Because of the difficulties with the manual translation process, at least two research teams evaluated the feasibility of automatic encoding systems. Tu et al. [8] applied natural language processing to transform 60 eligibility criteria into ERGO expressions that were valid for 70% of the criteria. Their method did however require manual pre-processing. Lonsdale [10] applied syntactic parsing and semantic conversion to transform 1545 eligibility criteria into predicate logic expressions that were subsequently mapped to the hospitals data dictionary and translated to Arden Syntax. They succeeded in generating queries for 520 (34%) criteria, though only half of these queries proved to be completely correct or yield useful information.

When it comes to transferring a CTRSS from the site of development to another hospital, the main barriers are due to differences in their information systems. Eligibility rules developed in one hospital usually do not fit the EHR of another because they make ‘*certain assumptions about the patient information model and terminology’*[8] and because of different documentation conventions. Therefore, the transfer of most of the existing CTRSS to other hospital settings requires special knowledge and extensive manual labor.

### Objectives

In summary, the necessity to translate eligibility criteria from free text into decision rules that are compatible with the EHR data constitutes the main challenge when developing and introducing CTRSS. An alternative to this rule-based approach is case-based reasoning, which relies on a knowledge base of past cases rather than explicit rules [11]. In the context of recruitment, a CTRSS based on case-based reasoning could dispense with the need for translating eligibility criteria and could also be implemented independently from the terminology of the EHR’s database. In this research, we implement such a CTRSS using predictive modeling algorithms in order to assess its feasibility for three clinical trials.

## Methods

At Erlangen University Hospital, patient facts from the 21 most important clinical desktop applications are transferred to a relational data warehouse (DWH) on a daily basis [12]. Patient age and gender are available from a relational table, while diagnosis and procedure codes are saved in entity-attribute-value (EAV) models. The code system used for diagnosis codes is the ICD-10 German Modification. For procedures, the ‘Operationen- und Prozedurenschlüssel’ (OPS) catalogue, a German modification of the International Classification of Procedures in Medicine (ICPM), is used. The DWH can be queried for clinical data of a patient using SQL. The cost-free statistics software R version 2.14 was used for fitting and evaluating the predictive models [13]. An off-the-shelf desktop computer was used to run the software.

We suggest that in a productive environment, case-based CTRSS would work as follows. When a new trial is to be supported by the CTRSS, a set of patients is manually screened. This could be either a one-time assessment of a set of past patients or a continuous screening process of patients as they enter the clinic. The eligibility of each patient is documented in the EHR. When a certain number of patients have been manually screened, the clinical data of eligible and ineligible patients is extracted from the EHR and used to derive a prediction model. The model is saved and can be applied to predict the eligibility of yet unscreened patients. The system thus proposes a list of trial participants who are subsequently manually assessed and their real eligibility entered in the EHR. With increasing numbers of assessed patients, the model can be continuously regenerated to improve its performance and the manual screening can be reduced.

In order to evaluate the feasibility of this CTRSS, we chose three clinical trials from different clinical departments of University Hospital Erlangen, which are briefly presented in Table 1. The trials were chosen because each featured a comparatively large number of local participants and a thorough screening process. The target of trial A was to compare standards of peri-operative care and outcome in 28 European countries [14]. Trial B validated the ability of different biomarkers to predict colorectal cancer stage, survival and response to chemo- and radiotherapy. Finally, trial C investigated the effects of adding oxaliplatin to standard combined modality treatment for rectal cancer [15]. After obtaining approval from the data security officer and confirmation from the ethical review board that no formal vote was necessary for our research, the principal investigator of each trial was asked to provide a list of all patients included in the trial until September 2012.

**Table 1:** Brief description of the three clinical trials used to evaluate the feasibility of predictive modeling for eligibility screening

**Abbreviation**	**Trial A**
**Full name**	European Surgical Outcome Study
**Recruitment period**	1 week, April 2011
**Department**	Department of Anesthesiology
**Inclusion criteria**	1. older than 16 years
	2. admitted for elective or non-elective inpatient surgery
**Exclusion criteria**	1. patients undergoing planned day-case surgery
	2. patients undergoing cardiac surgery, neurosurgery, radiological or obstetric procedures
**Abbreviation**	**Trial B**
**Full name**	Sensitive polyprobe approach for improved prediction of therapy response and assessment of prognosis in colorectal cancer
**Recruitment period**	3 years, December 2009 – December 2012
**Department**	Department of surgery
**Inclusion criteria**	1. initial diagnosis of histopathologically confirmed colorectal cancer of UICC stage I-IV
**Exclusion criteria**	1. inflammatory bowel disease (Crohn’s disease, Ulcerative colitis)
	2. hereditary tumor syndromes like Familial Adenomatous polyposis (FAP), Hereditary nonpolyposis colorectal cancer (HNPCC)
**Abbreviation**	**Trial C**
**Full name**	Prospective Randomized Multicenter Phase-III-study: Preoperative Radiochemotherapy and Adjuvant Chemotherapy With 5-Fluorouracil Plus Oxaliplatin Versus Preoperative Radiochemotherapy and Adjuvant Chemotherapy With 5-Fluorouracil for Locally Advanced Rectal Cancer
**Recruitment period**	3.5 years, July 2006 – February 2010
**Department**	Department of Radiation Oncology
**Inclusion criteria**	1. aged 18 years or older
	2. histopathologically confirmed rectal carcinoma with an inferior margin no more than 12 cm above the anal verge, as assessed by rigid proctoscopy
	3. evidence of perirectal fat infiltration (cT3–4) or lymph-node involvement (cN+), as assessed by endorectal ultra sound, multislice CT, or MRI
	4. Eastern Cooperative Oncology Group (ECOG) performance status 2 or lower
	5. adequate hematological, liver, and renal function
**Exclusion criteria**	1. metastatic disease
	2. prior radiotherapy or chemotherapy
	3. other cancers
	4. pregnancy or lactation
	5. clinically significant cardiac disease
	6. known peripheral neuropathy

UICC = Union for International Cancer Control, CT = X-ray computed tomography, MRI = Magnetic resonance imaging, CT3-4, cN+ = cancer stages according to TNM staging system.

For each clinical trial, an SQL statement was used to retrospectively query the DWH for the clinical data of all patients treated by the corresponding department during the trial’s recruitment phase. The query results were anonymised, written to a text file and passed to R for further processing. To increase the general validity of our evaluation, we restricted the data to those elements which are found in virtually every hospital information system: patient age and gender, as well as diagnosis and procedure codes.

Since the DWH stored patient data in an EAV schema, while predictive modeling algorithms as implemented in R packages expect these attributes in the individual columns of a tabulated dataset, an R function was deployed to convert between these two storage models. One attribute was added for each possible value of the diagnosis and procedure codes. Each attribute could assume one of two values: Y if the code was ever found documented for the patient and N if it was not. For example, if a patient had the diagnosis code ‘A00.0’, he would have a value of ‘Y’ in the column named ‘A00.0’.

Based on the review of predictive data mining algorithms in clinical medicine by Bellazzi and Zupan [16] we chose to compare the performance of the following algorithms for predictive modeling: decision trees based on the *CART* algorithm (as implemented in the R package *rpart*) [17], random forests by using decision trees in conjunction with bootstrapping (resampling), logistic regression with and without stepwise variable selection (*step* function) and support vector machines (R package *e1071*) [18].

Model fits were compared for different predictive algorithms and parameter configurations by plotting receiver operating characteristic (ROC) diagrams and assessing the area under the curve (ROC-AUC) in a separate subset of patients (*holdout method* with a test sample that consisted of 33% of all patients). The ROC curve represents all possible combinations of specificity and sensitivity for each predictive model, so ROC-AUC measures its overall performance. The theoretical maximum ROC-AUC value assumes 1 in case of perfect sensitivity and specificity, while non-informative (random) predictions would let ROC-AUC values converge to 0.5. ROC-AUC values were complemented with 95% confidence limits by generating 1,000 bootstrap resamples from each set of prediction-observation-pairs and subsequently invoking the *quantile* routine in conjunction with the resulting distribution of ROC-AUC values and probability parameters of 2.5% and 97.5%.

Algorithms which require a smaller training set to achieve a certain discriminatory power are preferable insofar as they permit replacing the manual screening process earlier. To gauge the dependency of system performance on the size of the training set, we progressively reduced the training data by iteratively discarding subsets from the training sample and refitting predictive models. With each iteration, approximately 30% of the patients were randomly selected and removed from the remaining training set.

Diagnosis and procedure codes are not independent from each other, but adhere to a mono-hierarchical structure. Different levels of this hierarchy can be relevant to decide between eligible and ineligible patients. Pregnant patients, for example, are usually excluded from clinical trials. During their stay at the hospital, this patient group is likely to obtain one or more of 459 different ICD codes beginning with the letter O. Unfortunately the standard algorithms for predictive modeling lack the ability to take such intrinsic connections between individual codes into account. The size of the training set necessary to identify for example a reproducible correlation between patient eligibility and one of 459 individual attributes in chapter O of the ICD catalogue could be substantially larger than that necessary to find a correlation with the aggregated attribute “catalogue chapter O”. Thus we assumed that aggregating diagnosis and procedure codes could improve the predictive quality of the models.

To test this hypothesis, we aggregated all diagnosis and procedure codes in three steps: no aggregation leaves all occurring codes as originally documented, category-level aggregation reduces all codes to the third hierarchy level of the corresponding catalogue and block-level aggregation reduces all codes to its second hierarchy level. For example, the procedure code 5–121.1 (incision of cornea with laser) and the diagnosis code L40.4 (Guttate psoriasis) would be reduced to 5–12 (corneal surgery) and L40 (Psoriasis) by category-level aggregation and to 5–08…5-16 (eye surgery) and L40…L45 (Papulosquamous disorders) by block-level aggregation. A patient was deemed affected by one of the superordinate diagnostic or procedural classes if any of the subordinate codes had been assigned to her.

For performance reasons, the number of diagnosis and procedure codes passed to the predictive modeling algorithms was limited following the aggregation process. Two methods to reduce the number of codes were compared: (1) taking the most frequently documented codes and (2) taking those codes which were most strongly associated with trial eligibility as measured by the chi-squared test, or alternatively by Fisher's exact when the data did not meet the frequency requirements for the chi-squared test. All models were calculated for the first 20 and 40 codes selected by each method.

## Results

When trial A was conducted previous to this study, 361 (70.6%) of 511 treated patients were manually included. In the same way, 320 (3.9%) of 8170 patients visiting the surgery department were included in trial B and 87 (1.6%) out of 5573 patients visiting the radiation oncology were included in trial C. The diagnosis and procedure codes which we extracted from the EHR assumed 2038 and 1648 (trial A), 5954 and 5816 (trial B) and 5052 and 5281 (trial C) different values respectively. The number of distinct codes was similar for all three trials after category-level aggregation (A: 1028, B: 1624, C: 1491 different codes) and block-level aggregation (A: 277, B: 302, C: 294 different codes). Each distinct code was represented by one column in the data set. Gender and age were available for all patients. Patients without any diagnosis or any procedure code were the exception. Most patients had several codes, although taken as a whole the data tables were sparsely populated. Each of the distinct diagnosis and procedure codes was documented for a median of 0.4% of all patients. The fraction increased to 1.6% after category-level aggregation and to 6.6% after block-level aggregation. The numbers for all three trials are summarized in Table 2.

**Table 2:** Data volume for the three clinical trials: number of patients treated by the department during the recruitment period, number of patients included, number of attributes documented for all these patients and the fraction of attributes with a documented value for each patient

**Trial**	**Patients treated**	**Patients included**		**Number of attributes [n]/Valued cells [%] (target variable, age, gender + no. of codes)**			**Patients without any**	
	**[n]**	**[n]**	**[%]**	**No aggregation**	**Category-level aggregation**	**Block-level aggregation**	**Diagnosis**	**Procedure**
A	511	361	70.6	3,689/0.7	1,031/1.9	280/5.6	3	6
B	8,170	320	3.9	11,773/0.3	1,627/1.6	305/6.6	6	215
C	5,573	87	1.6	10,336/0.4	1,494/2.1	297/8.1	8	0

Each different diagnosis and procedure code was treated as an independent patient attribute.

Trial A excluded patients undergoing obstetric procedures from inclusion. The 20 diagnosis and procedure codes identified as most strongly associated with patient eligibility mainly reflect this. For example, the original diagnosis codes Z37.0 (single live birth), O82 (caesarean section) and O09.6 (full term pregnancy) were included in building the predictive model based on the originally documented codes. After block-level aggregation, ICD codes for complications during pregnancy or birth (O20…O29, O85…O99) and the OPS codes for obstetric procedure (5-72…5-75) were additionally incorporated in the models. However, the strong association of some codes like 5–50 (liver surgery) with eligibility is less obvious, as it is not explicitly mentioned in the trial’s eligibility criteria. Liver surgery is one of the procedures not excluded by the inclusion criteria of the trial. Patients with a liver surgery were thus found to be more common in the set of trial participants than in the set of non-participants and the attribute is therefore associated with trial eligibility. The lists showing the 20 attributes most associated with eligibility are given for all trials and aggregation levels in Additional file 1.

**Figure 1:**
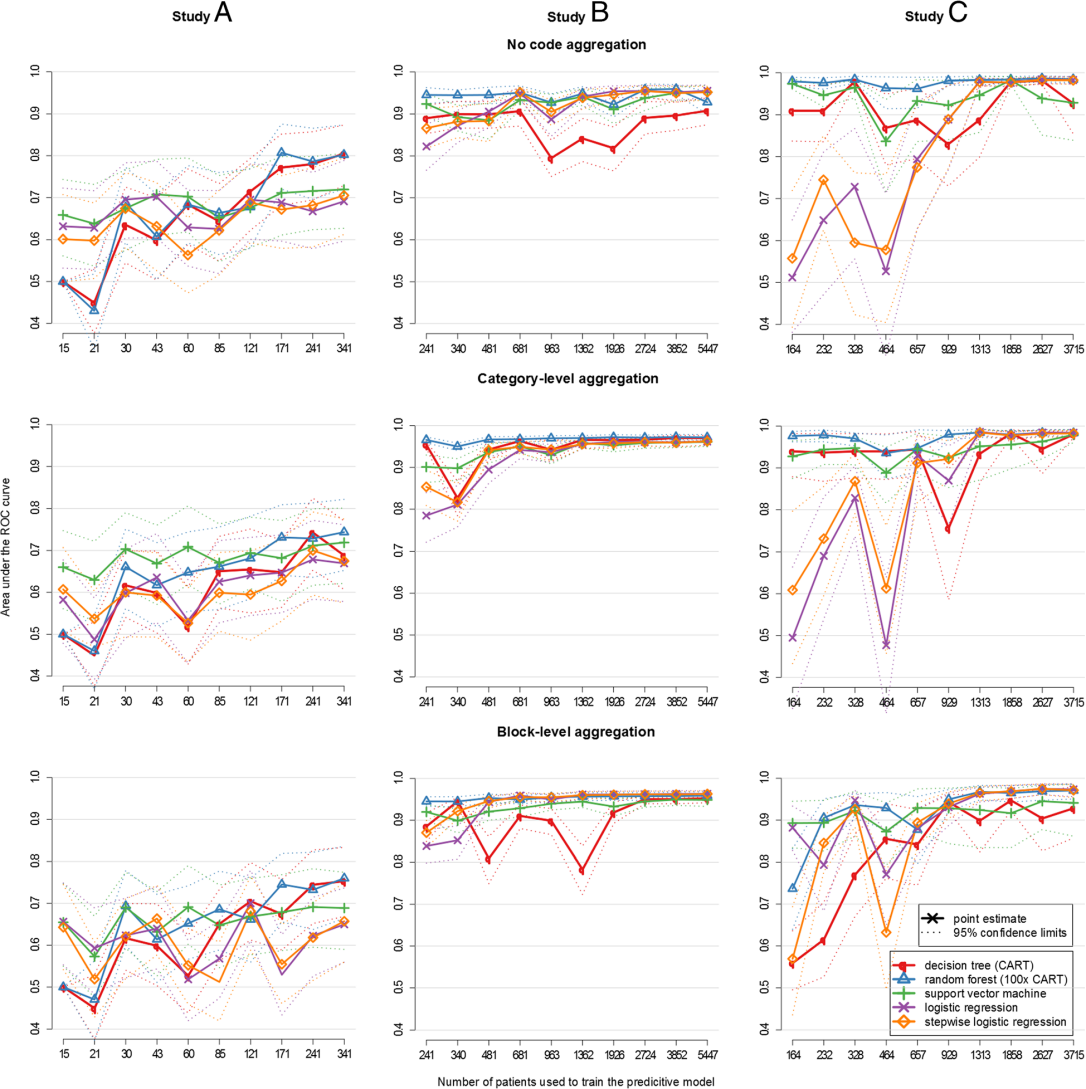
**Evaluation of the prototype’s performance for models based on age, gender and the 20 codes most associated with trial eligibility: model fits were compared by area under the receiver operating characteristic (ROC) curve complemented with 95% confidence limits (y-axis).** Models were trained with differently sized training sets of patients (x-axis). All models were tested against one third of the original patient set (hold-out method), thus the maximum size of the training set was two thirds of all patients. The experiment was done for the original codes as documented, after aggregation to category level and after aggregation to block level.

The ROC-AUC values generally improved with increasing sizes of the training set. Neither category-level nor block-level aggregation of diagnosis and procedure codes led to a consistent influence on any of the models based on the codes most associated with eligibility. Selecting the 20 most associated diagnostic codes generally outperformed models based on the 20 most frequent codes, but the differences decreased with increasing code aggregation. Furthermore, there appeared to be no notable difference between models based on 20 and 40 codes. Figure 1 shows the ROC-AUC values for all 450 models based on the 20 most associated codes. The figures for the corresponding models based on the 40 most associated and on the 20 most frequent codes can be found in Additional file 2: Figure S2 and Additional file 3: Figure S3 respectively.

The following numbers are given for the best performing models, which were based on the most associated codes without aggregation. After learning from 171 patients of trial A (33% of all patients), ROC-AUC reached a maximum of 0.81 (CI: 0.72-0.88) for random-forest-based predictions and did not increase further with larger training sets. For smaller training sets, support vector machines performed better than random forests, achieving ROC-AUC values of up to 0.71 (CI: 0.61-0.79) with a 60 patients (12%) training set. For trial B and C, all algorithms showed high maximum ROC-AUC values of up to 0.96 (CI: 0.95-0.97) and 0.99 (CI: 0.98-0.99) respectively. Random forests and support vector machines reached maximum performance early after learning from only approximately 3% of all patients screened for trials B and C, corresponding to 241 and 164 patients respectively. This would correspond to 1 and 1.3 months of manual screening of all admitted patients. Decision trees and logistic regression models were susceptible to small training sets and reached the other algorithms’ ROC-AUC values only after including approximately 20% of all patients. The complete numeric results including confidence intervals are given in Additional file 4.

## Discussion

The objective of this research was to evaluate the feasibility of using predictive modeling for the identification of potential participants of clinical trials. The major advantage compared to the currently predominant rule-based approaches lies in the fact that the trial’s eligibility criteria do not need to be translated into a computable form. This allows the proposed system to be independent of the concrete representation of clinical data in a specific hospital and thus to be easily applicable across institutions. Furthermore, it capitalizes exclusively on routine documentation and offers extensive scope for automation.

Our results indicate that case-based CTRSS can be suitable for practical implementation in clinical trials. Predictive models based on random forests were generally among the best performing algorithms with ROC-AUC values of up to 0.8 for one and of more than 0.95 for the other two of three exemplary trials. The full potential of this algorithm was reached for all three trials after learning of about 200 manually screened patients (eligible and ineligible). Thus the fraction of patients who need to be screened manually is high for trials with a low total number of patients to screen (33% in trial A) and low for trials that require mass screening (3% in trial B and C). For the latter, we believe our approach to constitute a viable alternative to explicitly modeling EHR specific rules for inclusion and exclusion criteria. In addition to the size of the training set, theoretical considerations indicate that the predictive performance should also depend on the ratio of eligible to ineligible patients. For example, if we considered the extreme case of some hypothetical cohort with no (or all) patients eligible, no associations between eligibility and other attributes would be observable and could thus be included within the predictive models. This suggests that training sets with a more balanced distribution of eligible and ineligible patients, all other things equal, should gradually allow training better-discriminating predictive models. Within our limited set of three investigated studies, however, other determinants such as the absolute size of the training sample appear to have been more important.

We believe that the size of the training data set required to obtain a useful predictive model is mainly dependent on the properties and number of attributes which describe the patients, but independent from the number of patients required for the trial. Adding attributes can increase or decrease the size of the training set that would be necessary to attain the desired predictive ability. If additional attributes are related to patient eligibility, they would most likely contribute to decrease the number of patients that require manual screening. Otherwise, these additional attributes merely add noise (i.e. irreproducible associations) to the dataset that must be accommodated by a larger set of training data. The relationships between attribute selection and system performance will be object to further investigation.

The right balance between sensitivity and specificity of a CTRSS is trial-specific. Sensitivity will be favored if the population of eligible patients is small, while a high specificity will be paramount, if the population is large [19]. Predictive modeling provides some flexibility with respect to choosing among specificity and sensitivity constellations by adapting probability thresholds according to trial requirements. When sensitivity is important, specificities of 0.25 [20] and 0.31 [19] may still be perceived positively by their users. When the focus lies on specificity and the trial’s eligibility criteria correspond well to the data elements in the EHR, rule-based CTRSS achieve a high specificity of up to e.g. 0.84 [21] and 0.96 - 1.0 [22]. For trials B and C the prototype’s performance was similar to that of these rule-based systems. For trial A however, the ROC curves for the best performing predictive models allow only for a specificity of about 0.4 for a fixed sensitivity of 1 after learning from one third of all patients. In contrast, an SQL-based CTRSS, which was actually used to conduct this trial at Erlangen University Hospital achieved nearly 100% sensitivity and specificity and could be employed from day one [23]. On the one hand, the rule-based CTRSS profited in this instance from a most favorable choice of eligibility criteria that could be matched exactly to EHR data elements. On the other hand, the predictive modeling approach suffered from the relatively small size of the learning set for trial A.

Our evaluation study is limited in that only a selected subset of the available patient attributes was used to derive the prediction models. While additional parts of the clinical documentation such as laboratory results may also be usable in this context, we intentionally restricted our analysis in order to obtain models that are primarily straightforward and generalizable insofar as they build on highly uniform data. The resulting models can obviously reflect the original eligibility criteria only to the extent that there are corresponding associations in the data. In principle though, every hospital can incorporate data elements from its proprietary documentation, for example laboratory, medication and assessment form data, to increase the system’s accuracy. This would not require any modification in the method for data analysis and preparation other than the inclusion of additional data tables from the EHR.

Though the early results of the prototype are encouraging, we see a number of potential enhancements that future research could investigate. First, the temporal aspects of and relationships between patient attributes were not regarded during data collection and the modeling process. Incorporating temporal information could be important for a subset of clinical trials that require events to appear in a specific order or in a given timeframe. Second, free text data have been shown to contain information that is relevant for the purpose of eligibility assessment [24-26]. So far the prototype only supports numeric attributes and attributes with pre-defined value lists. Zhang et al. recently reported on their subtree match method, which finds structural patterns in free text sentences and thus allows to find similar sentences in other documents [27]. Both the list of keywords and their grammatical structure can be automatically derived from the text. We believe this approach could be further enhanced to allow the inclusion of small text fragments into the predictive modeling process. Third, both the case-based and the rule-based CTRSS are heavily dependent on the completeness and correctness of the EHR’s clinical data. Further studies should also compare the robustness of case-based and rule-based CTRSS for missing data and incorrect data. We hypothesize that case based systems are more insensitive to missing data, because they are based on a broader base of data elements and more sensitive to incorrect data as these cause imprecise prediction models.

Within multicenter trials, the prediction model generated by one site could be distributed to the others only if they share a common terminology. For example, the models developed in this feasibility study are restricted to hospitals using the ICD-GM and OPS catalogues. The wish to share the same predictive model with each other would inhibit all participating institutions to use the wealth of potentially relevant patient data encoded in a proprietary terminology. This dependency on EHR terminologies is however not restricted to case-based reasoning, but restricts the distribution of rule-based CTRSS and clinical decision support systems in general in the same way [28]. Including institution-specific attributes in the analysis will generally be pursued to optimize the performance of the CTRSS. In this case, predictive learning has an important advantage over explicit rules: new institution-specific prediction models can be trained automatically without having to analyze the EHR data elements regarding their meaning and usage by the intervention clinic in order to specify the relevant data elements. While the meaning of the data is of decisive importance where rules are to be developed, it is irrelevant to train the predictive model. Additionally, the setup of the case-based system itself requires only the extraction and formatting of all available patient data from the EHR.

## Conclusions

Case-based reasoning constitutes a viable alternative to the widely applied rule-based approach to reusing medical records for patient recruitment and can be successfully implemented using predictive modeling algorithms. For two out of three evaluated trials system performance was comparable with results published for rule-based systems even though a technically simple approach was used in the prototype. The case-based CTRSS offers many advantages, namely its independence from the trial protocol’s definition of eligibility criteria and from the terminology of the clinical database. Future investigations might delineate advantages and disadvantages of the approach when compared to rule-based methods in particular.

## Abbreviations

EHR: Electronic health record; CI: Confidence interval; CTRSS: Clinical trial recruitment support system; SQL: Standard query language; DWH: Data warehouse; EAV: Entity-attribute-value; OPS: Operationen- und Prozedurenschlüssel; ICPM: International Classification of Procedures in Medicine; ROC: Receiver operating characteristic; ROC-AUC: Area under the ROC curve.

## Competing interests

The authors declare no competing interests.

## Authors’ contributions

FK conceived of the study and drafted the manuscript. DL and RF selected and conducted case trial 1, acquired the data and accompanied data analysis. AS and CN selected and conducted case trial 2, acquired the data and accompanied data analysis. RC and MS selected and conducted case trial 3, acquired the data and accompanied data analysis. HP contributed to the design of the study and helped draft the manuscript. DT designed the study, executed the statistical analysis and helped draft the manuscript. All authors reviewed and approved the manuscript.

## Pre-publication history

The pre-publication history for this paper can be accessed here:

http://www.biomedcentral.com/1472-6947/13/134/prepub

## Supplementary Material

Additional file 1 20 attributes most associated with eligibility for all trials and aggregation levels. Click here for file

Additional file 2: Figure S2 Graphical results for models trained with the 40 diagnosis or procedure codes most associated to trial eligibility. Click here for file

Additional file 3: Figure S3 Graphical results for models trained with the 20 diagnosis or procedure codes most associated to trial eligibility. Click here for file

Additional file 4 Numerical results for all models including confidence intervals. Click here for file
