# Supplementary material for: Evaluating predictive modeling algorithms to assess patient eligibility for clinical trials from routine data
Source: BMC Med Inform Decis Mak. 2013 Dec 9;13:134. doi: 10.1186/1472-6947-13-134 (PMC4029400; doi:10.1186/1472-6947-13-134)
Supplement: Additional file 3: Figure S3 — Graphical results for models trained with the 20 diagnosis or procedure codes most associated to trial eligibility. [file 1472-6947-13-134-S3.pdf]

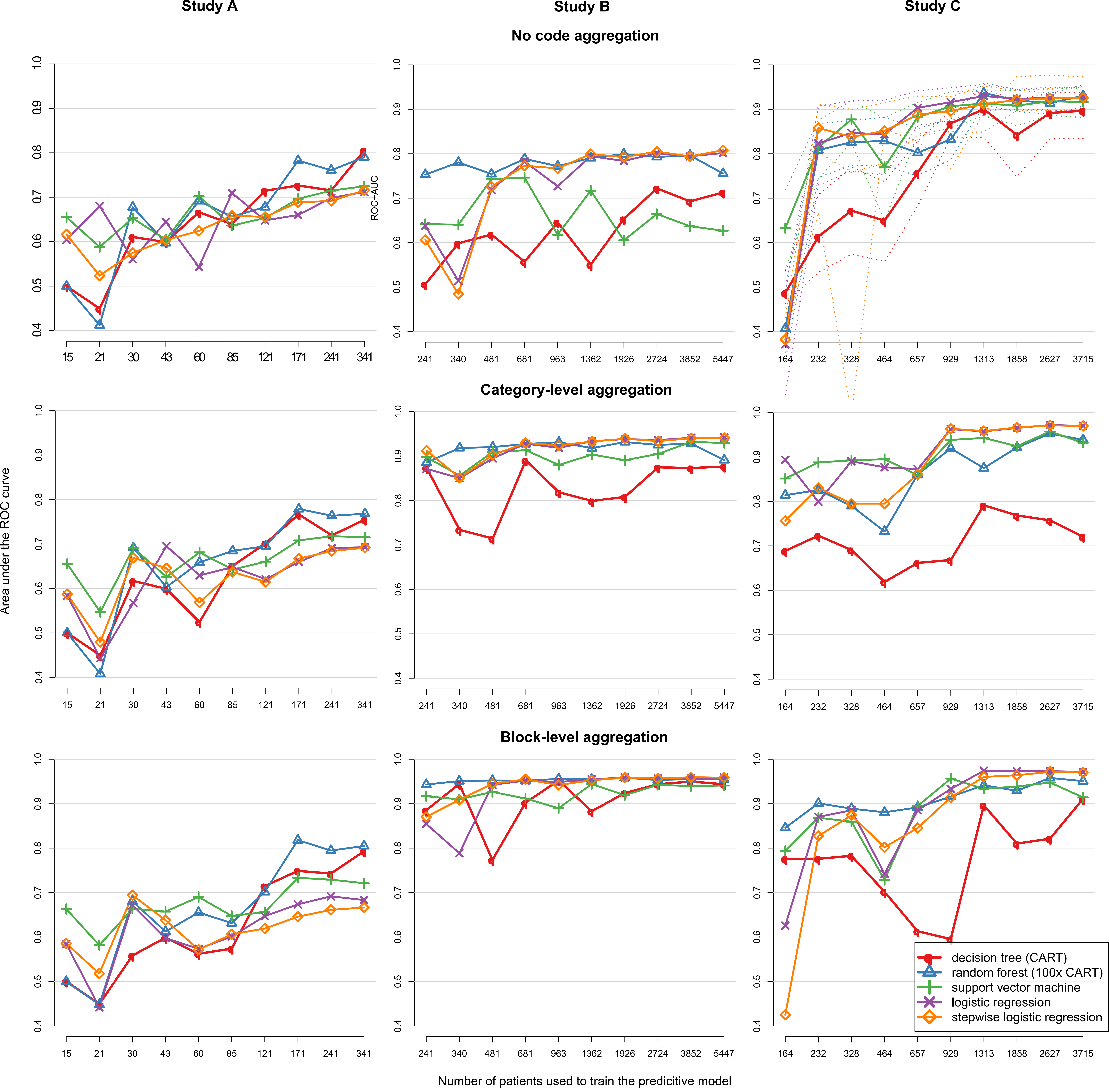

Supplement 3: Models trained with the 20 diagnosis or procedure codes most often documented for all patients of the clinic
